# Traumatic diaphragmatic rupture with delayed gastric incarceration

**DOI:** 10.4103/0974-2700.66539

**Published:** 2010

**Authors:** Nisar Ahmad Wani, Tasleem Lone Kosar, Asrar Ahmad, Mohammad Yusuf

**Affiliations:** Departments of Radiodiagnosis and Imaging, Srinagar, J & K, India; 1 Cardiovascular Thoracic Surgery, Srinagar, J & K, India; 2 Gastroenterology, Sher-I-Kashmir Institute of Medical Sciences (SKIMS), Srinagar, J & K, India

Sir,

Penetrating thoracoabdominal trauma, like gunshot and stab injury, is associated with a high likelihood of rupture of the diaphragm, which is more common on the left side.[1] Such a diaphragmatic injury is commonly asymptomatic and does not acutely manifest any specific physical findings or radiographic abnormalities. However, diaphragmatic hernias may present later with some delay between trauma and diagnosis.[1,2] Post-traumatic diaphragmatic hernias may be complicated by obstruction, strangulation and perforation of the stomach or intestine within the chest.[3] Multidetector-row computed tomography (CT) (MDCT) enhances the diagnosis of post-traumatic diaphragmatic defects with associated complications.[4] However, penetrating lower chest injuries should be evaluated with laparoscopy or thoracoscopy in order to identify diaphragmatic injuries early and avoid later complications.[5]

**Figure 1:**
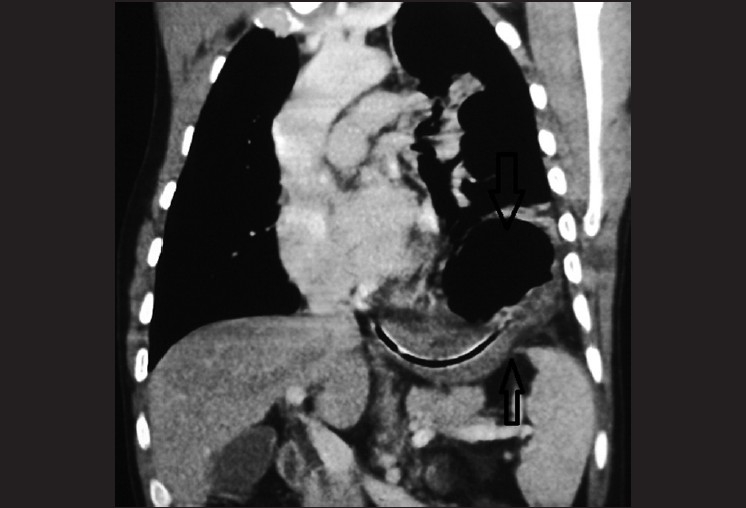
Coronal reformat computed tomographic image showing a defect in the left hemidiaphragm with the stomach containing the nasogastric tube within the defect (upward arrow), with the stomach being dilated superior to it inside the left hemithorax (downward arrow)

We describe a case of delayed herniation of the stomach with incarceration, following penetrating trauma to the left lower chest. A 40-year-old man presented to the emergency department with acute-onset epigastric pain and vomiting from the last 5 days. The patient reported an episode of stab injury on the left side of the lower chest 1 month back, which was managed with intercostal tube thoracostomy. On physical examination, no significant findings were elicited. A nasogastric tube was placed and an urgent X-ray of the chest, including both domes, was performed. The tip of the nasogastric tube within the stomach was displaced superiorly just underneath the apparently elevated left hemidiaphragm; mediastinum was displaced toward the right. MDCT was performed, which showed left pleural effusion with the stomach containing the nasogastric tube within the left hemithorax [Figure 1]. The sagittal and coronal reformations revealed a defect in the left hemidiaphragm posterolaterally, containing a narrow segment of stomach with a greater dilated portion within the chest superior to it [Figure 1]. Thus, the diagnosis of delayed diaphragmatic hernia with incarceration of the stomach was confirmed. An immediate thoracotomy was performed and the stomach repositioned back into the abdomen followed by repair of a 3 cm rent in the left hemidiaphragm.

Early diagnosis of the post-traumatic diaphragmatic rupture in penetrating thoracoabdominal trauma is crucial to avoid delayed presentation with hernia and complications.[1,2] This may be accomplished by laparoscopy in patients in whom immediate laparotomy is not indicated at the time of injury.[2,5]

When the early phase is over and the patient presents with upper abdominal pain, a history of recent or remote penetrating thoracoabdominal trauma on the left side should give rise to clinical suspicion of a delayed diaphragmatic hernia complication like incarceration or strangulation of the herniated stomach.[3] Proper radiological examinations will help in diagnosis.[2] Chest X-ray is the initial radiologic investigation, which reveals absence of fundic gas in its normal position, elevation of the hemidiaphragm with its absent sharp outline and coiled nasogastric tube located within the left hemithorax. Oral administration of barium or water-soluble contrast may enhance the detection of bowel within the chest.[2] MDCT is the most appropriate second-line study.[2,4] Multiplanar reformations of high spatial resolution improve the accuracy of CT in demonstrating diaphragmatic defects and hernias. The demonstration of conclusive radiologic findings of collar sign as a constriction of hollow viscus at the diaphragmatic defect and dependent viscera sign as abdominal organs set against posterior ribs enhances the diagnosis of post-traumatic diaphragmatic hernia.[4] The management strategy includes hernia reduction, pleural drainage and repair of the diaphragmatic defect.[3]
